# InsR/FoxO1 Signaling Curtails Hypothalamic POMC Neuron Number

**DOI:** 10.1371/journal.pone.0031487

**Published:** 2012-02-02

**Authors:** Leona Plum, Hua V. Lin, Kumiko S. Aizawa, Yitian Liu, Domenico Accili

**Affiliations:** Naomi Berrie Diabetes Center, Department of Medicine, Columbia University Medical Center, New York, New York, United States of America; Institut de la Vision, France

## Abstract

Insulin receptor (InsR) signaling through transcription factor FoxO1 is important in the development of hypothalamic neuron feeding circuits, but knowledge about underlying mechanisms is limited. To investigate the role of InsR/FoxO1 signaling in the development and maintenance of these circuits, we surveyed the pool of hypothalamic neurons expressing *Pomc* mRNA in different mouse models of impaired hypothalamic InsR signaling. InsR ablation in the entire hypothalamus did not affect *Pomc*-neuron number at birth, but resulted in a 25% increase, most notably in the middle arcuate nucleus region, in young adults. Selective restoration of InsR expression in POMC neurons in these mice partly reversed the abnormality, resulting in a 10% decrease compared to age-matched controls. To establish whether FoxO1 signaling plays a role in this process, we examined POMC neuron number in mice with POMC-specific deletion of FoxO1, and detected a 23% decrease in age-matched animals, consistent with a cell-autonomous role of InsR/FoxO1 signaling in regulating POMC neuron number, distinct from its established role to activate *Pomc* transcription. These changes in *Pomc* cells occurred in the absence of marked changes in humoral factors or hypothalamic NPY neurons.

## Introduction

Hypothalamic neurons are targets of metabolic stimuli whose action is required to control feeding behavior and energy homeostasis. The pancreatic hormone insulin relays peripheral anabolic signals to the hypothalamic control center [1]. Insulin receptors (InsR) are widely expressed in the brain, with the highest concentrations in the olfactory bulb, hippocampus, cerebral cortex, cerebellum, and hypothalamus [2], [3]. Similarly, the insulin-regulated transcription factor FoxO1 has been detected in the olfactory cortex, striatum, hippocampus, amygdalo-hippocampal region, and hypothalamus [4], [5]. FoxO1 is present in a majority of cells in hypothalamic nuclei involved in energy homeostasis, such as the dorsomedial (DMH), ventromedial (VMH), and arcuate (ARC) hypothalamic nuclei. In the latter, FoxO1 is abundant in two neuronal subpopulations characterized by expression of pro-opiomelanocortin (POMC) or neuropeptide Y (NPY) and agouti-related protein (AgRP) [5].

Metabolic and/or energy-homeostatic effects of altered activity of various components of the InsR/FoxO1 signaling cascade in hypothalamic AgRP or POMC neurons have been investigated in genetically modified animal models. While a role of InsR/FoxO1 signaling in the development of the hypothalamic feeding circuits and neurons in the ARC is well established [6], [7], [8], the underlying mechanisms are nebulous. Both human and experimental animal studies have investigated the developmental role of the intrauterine and early postnatal environments in this context, including maternal nutritional status, fetal insulin and leptin levels, and perinatal dietary environment [9], [10], [11], [12]. In particular, a genetic model of maternal insulin resistance and hyperinsulinemia is associated with increased *Pomc*-positive cell number in the offspring in the early postnatal period, suggesting the terminal differentiation, but not the ontogeny, of POMC neurons is sensitive to changes in ambient metabolic cues [13]. Moreover, a very recent study demonstrated that perinatal insulin deficiency causes altered POMC neuronal morphology in the offspring [14]. However, there are no reports on the role of InsR/FoxO1 signaling in the developing hypothalamic neurons *per se*.

It has been suggested that not only neuronal plasticity [15], but also *de novo* neurogenesis of hypothalamic neurons—including AgRP and POMC neurons—serve as mechanisms controlling energy balance in response to environmental and physiologic cues in the adult brain [16]. The *de novo* regenerative potential has been shown to arise from resident neural stem cells (NSCs) that contribute directly to neurogenesis throughout development and adulthood. In the adult brain, NSCs reside primarily in the subventricular zone (SVZ) of the lateral ventricles and in the subgranular zone of the hippocampal dentate gyrus. Consistent with its known function as a modulator of cell cycle, FoxO1 is abundant in NSCs of the SVZ. Even though somatic inactivation of FoxO1 in the mouse brain does not cause developmental abnormalities, it results in increased brain size in adult mice, partly due to increased proliferation of NSCs and/or progenitor cells in the brain of young adult mice [17]. In contrast, the NSC-independent effects of altered InsR/FoxO1 signaling on the functional anatomic features of these ARC neurons are unknown.

**Figure 1:**
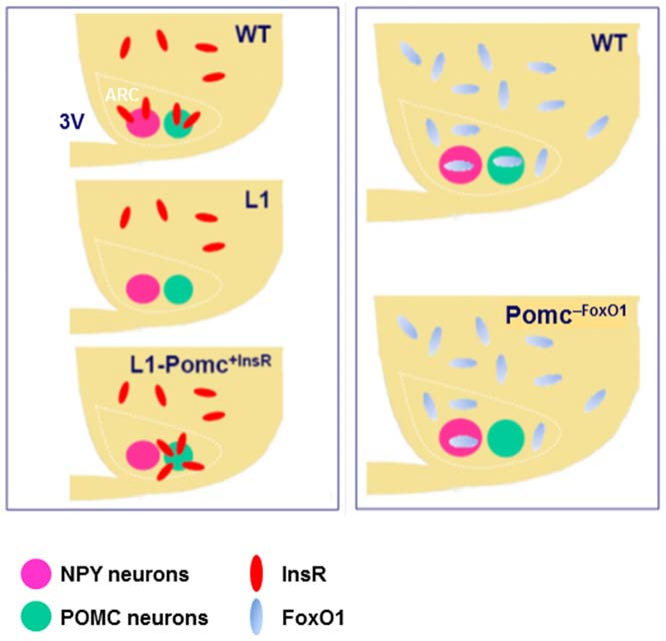
Schematic diagrams of the hypothalamus of mouse models used in this study. 3V, third ventricle; ARC, arcuate nucleus of the hypothalamus. Recombination in POMC neurons of *L1-Pomc^+InsR^* and *Pomc^–Foxo1^* mice is induced by a *Pomc-Cre* transgene.

In this study, we used neuron-specific knockout and reconstitution approaches to investigate the role of cell-autonomous signals mediated by InsR and FoxO1 in the development and maintenance of hypothalamic POMC neurons. To this end, we used three mouse models: (*i*) L1 transgenic/knockout mice express InsR in liver, pancreatic beta cells, and most neurons outside the hypothalamus, but lack InsR in the hypothalamus, including ARC AgRP and POMC neurons [7], [18]; (*ii*) mice lacking InsR in all hypothalamic neurons except POMC neurons (*L1-Pomc^+InsR^*) to distinguish cell-autonomous from non-autonomous effects of InsR signaling in ARC POMC neurons (**Figure 1**). Finally, we analyzed the contribution of FoxO1 signaling to POMC neuronal development, using (*iii*) mice with POMC neuron-specific FoxO1 ablation (*Pomc^–Foxo1^*). We demonstrate that insulin curtails the number of functional ARC POMC neurons in young adult mice via cell-autonomous InsR/FoxO1 signaling, but has no effect on prenatal and pre-pubertal development. Our data indicate that InsR/Foxo1 signaling regulates POMC neuron function by controlling their number between puberty and young adulthood.

## Results

### InsR deficiency results in increased POMC neuron number in the ARC of L1 mice

To investigate the contribution of InsR signaling to development and maintenance of ARC POMC neurons, we determined *Pomc*-expressing cell number by fluorescent *in situ* hybridization (FISH) in the ARC in L1 mice. These mice have a >95% reduction of InsR levels in the ARC (**Figure 1**) and blunted insulin signaling in both hypothalamic cell extracts and ARC neurons *in vivo* [7], [18], [19].

POMC and NPY/AgRP neurons in the rodent ARC nucleus arise at embryonic day (e)11.5–12.5 [20], and acquire their definitive phenotype mainly within the second and third postnatal week [21], [22], [23]. To determine whether development of *Pomc* neurons requires InsR signaling, we counted ARC POMC neurons at postnatal day (P) 7, and found them to be comparable between L1 and WT control mice (**Supplementary Figure S1A**), indicating that hypothalamic InsRs are dispensable for generation of *Pomc*-expressing ARC neurons. We obtained a similar result when we determined the number of *Pomc*-positive cells after completion of differentiation, but before the onset of puberty, in five week-old mice (P35) (**Supplementary Figure S1B**). This finding suggests that POMC neurons are capable of developing their terminal peptidergic phenotype despite the absence of InsR.

**Figure 2:**
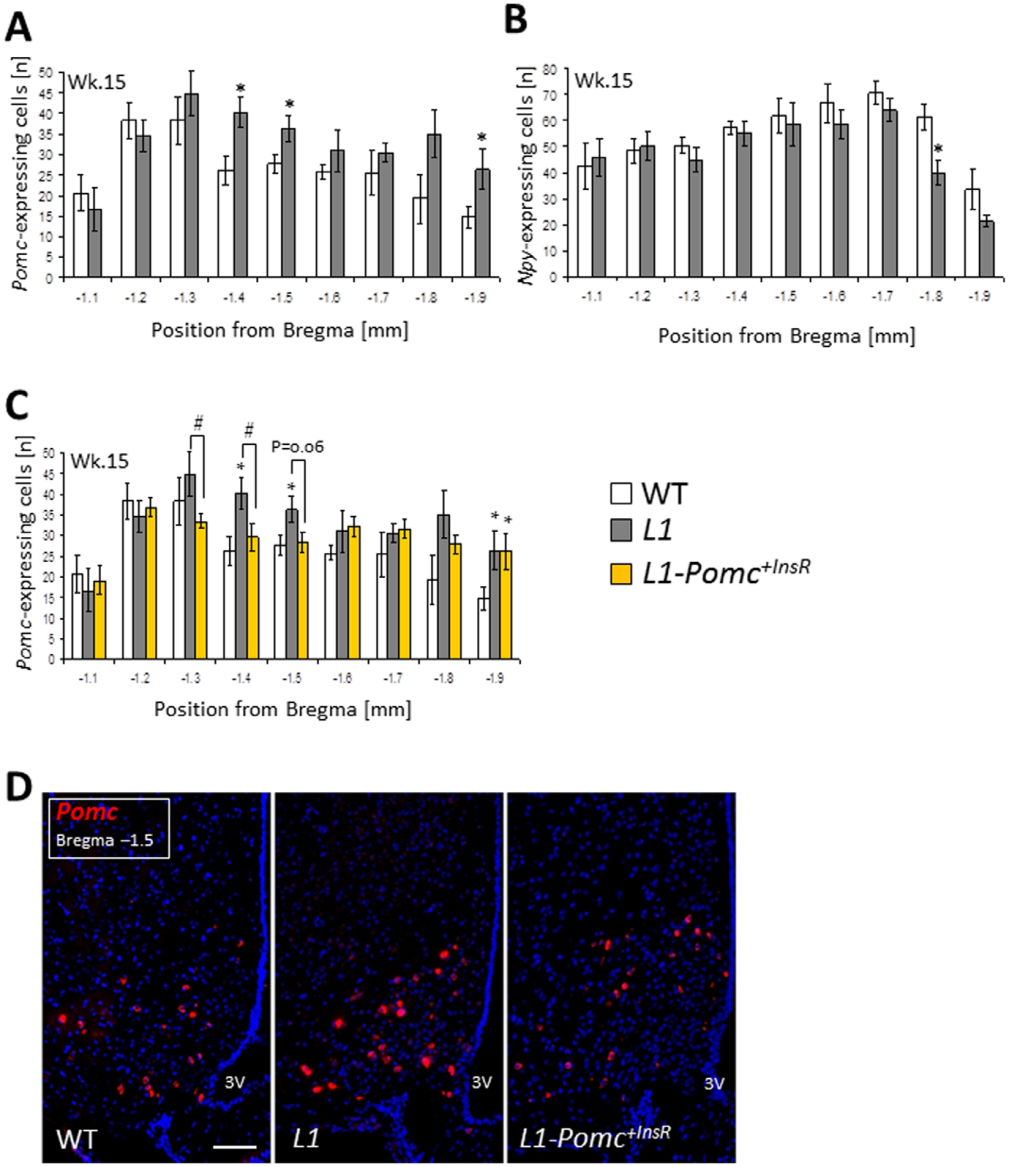
InsR affects *Pomc* but not *Npy* neuron numbers in adult L1 mice. Number of *Pomc*-expressing (**A**) and *Npy*-expressing (**B**) cells per ARC hemisection in L1 mice (grey bars, n = 5) and WT controls (white bars, n = 5) at the age of 15 weeks. Number of *Pomc*-expressing cells per ARC hemisection in the same mice as in A compared to *L1-Pomc^+InsR^* (yellow bars, n = 6) at the age of 15 weeks (**C**). Exemplary *Pomc* FISH pictures of WT, L1, and *L1-Pomc^+InsR^* mice (**D**). Scale bar is 100 µm. All data are means ± S.E.M.. *P<0.05 L1 *vs.* WT control. ^#^P<0.05 L1 *vs. L1-Pomc^+InsR^*.

By contrast, 15 week-old L1 males showed a 25% increase in the number of *Pomc*-positive cells compared to controls, with significantly increased *Pomc* neuron numbers in the middle ARC (**Supplementary Figure S2**, **Figure 2A**). To determine whether the increased *Pomc* neuron count reflects a post-pubertal increase of hypothalamic cells due to absence of InsR in most ARC cells or alternatively to a *Pomc* neuron-specific effect, we determined the number of NPY/AgRP neurons in adjacent ARC sections of the same mice. Quantification of *Npy*-expressing neurons in the ARC revealed similar numbers of these cells—especially in the middle ARC—in L1 mice and WT controls (**Figure 2B**), suggesting that the observed effect is specific to POMC neurons.

### Reconstitution of InsR attenuates raised POMC neuron numbers in L1 mice

The number of POMC neurons is affected by changes in ambient metabolic signals during development [13]. Thus, it cannot be excluded that the observed increase in *Pomc*-expressing neurons in adult L1 mice is merely a result of altered metabolism in L1 mice (e.g., glucose intolerance and hyperinsulinemia) rather than a direct consequence of hypothalamic InsR deficiency. However, two observations make this hypothesis unlikely: (*i*) POMC neuron development is seemingly unaffected by the absence of InsR before puberty and (*ii*) we only studied non-diabetic mice. Nonetheless, to distinguish between a POMC-specific, cell-autonomous effect of InsR signaling, and a cell non-autonomous effect, we asked whether restoration of InsR in POMC neurons would reverse this phenotype and normalize *Pomc*-positive cell number in L1 mice. Thus, we studied a model with reconstituted InsR expression and signaling in POMC neurons of L1 mice (*L1-Pomc^+InsR^*), generated using a conditional locus knock-in approach [7]. Adult *L1-Pomc^+InsR^* mice exhibit the same glucose-intolerant, hyperinsulinemic phenotype observed in L1 mice. Consistent with our hypothesis, 15 week-old *L1-Pomc^+InsR^* mice showed an approximately 10% reduction of total ARC *Pomc* neurons (**Supplementary Figure S2**), with a statistically significant decrease of *Pomc*-positive cells in the middle ARC (**Figure 2C, D**). Taken together, these data suggest that InsRs are dispensable for ontogeny and terminal differentiation of ARC POMC neurons, but play a role in the regulation of this neuronal pool size during adulthood.

### *Pomc*-specific ablation of FoxO1 reduces *Pomc* neuron numbers in adult *Pomc^–Foxo1^* mice

Transcriptional effects of insulin are mediated to a large extent through the forkhead transcription factor FoxO1. To test the hypothesis that alterations of InsR signaling with specific effects on POMC cell maintenance are conveyed by FoxO1, we studied mice in which FoxO1 is selectively deleted in *Pomc*-expressing cells in the ARC and pituitary (*Pomc^–Foxo1^*) [8]. In this model, ablation of FoxO1 mimics constitutive activation of InsR signaling at the transcriptional level.

Similar to the observation made in pre-pubertal L1 mice, *Pomc^–Foxo1^* mice exhibited unaltered *Pomc* neuron numbers at P7 and P35 as compared to controls (**Supplementary Figure S3**). This suggests that FoxO1 in POMC neurons, like InsR, is not required for development and differentiation of appropriate numbers of *Pomc*-expressing cells in the ARC.

**Figure 3:**
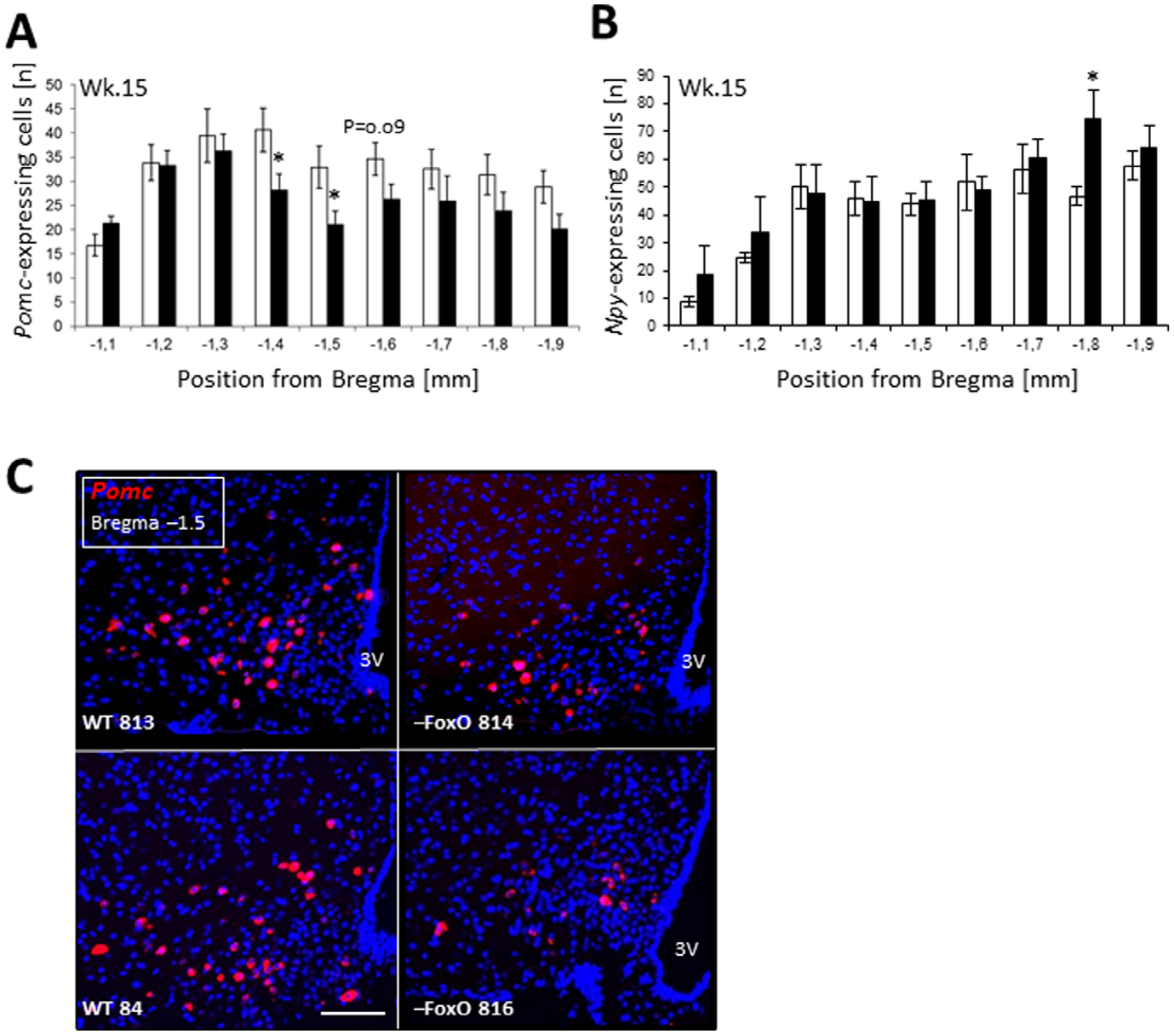
Reduced *Pomc* and unaltered *Npy* neuron number in adult *Pomc^–Foxo1^* mice. Number of *Pomc*-expressing (**A**) and *Npy*-expressing (**B**) cells per ARC hemisection in *Pomc^–Foxo1^* mice (black bars, n = 7–9) and WT controls (white bars, n = 7–9) at the age of 15 weeks. Exemplary *Pomc* FISH pictures of two WT and *Pomc^–Foxo1^* mice (**C**). Scale bar is 100 µm. All data are means ± S.E.M. *P<0.05 *vs.* WT control.

Consistent with our predictions, the number of *Pomc*-positive cells in the ARC of adult *Pomc^–Foxo1^* mice was reduced by 23%, as compared to WT controls (**Supplementary Figure S4**). This reduction in total *Pomc* count reflected a significant reduction of *Pomc*-positive cells in the middle ARC (**Figure 3A, C**). Furthermore, quantification of *Npy*-expressing neurons in the ARC showed that the decrease in cell count was specific to *Pomc*-positive cells and was not paralleled by decreased *Npy* cell counts in *Pomc^–Foxo1^* mice (**Figure 3B**). Of note, the anatomic region with markedly reduced *Pomc* counts in *Pomc^–Foxo1^* mice corresponded to the region with increased *Pomc* cell counts in L1 mice, suggesting that InsR/FoxO1-mediated signals are specifically required in this region to attain physiologic levels of functional POMC neurons (as assessed by *Pomc* expression).

## Discussion

Although compensatory changes in neuronal circuits controlling feeding and energy balance in response to environmental and pathophysiological cues are frequently observed, the underlying mechanisms remain incompletely understood. In addition to changes in neuropeptide expression and cellular electric activity, alterations of functional anatomic characteristics such as hypothalamic cell numbers and synaptic inputs may change in response to metabolic signals [13], [15]. The current study addressed the effects of insulin action on the development and maintenance of functionally active ARC POMC neurons in hypothalamic InsR-deficient L1 mice using *Pomc in situ* hybridization, which provides a similar assessment of POMC neuron number as a POMC-GFP reporter strain [24]. To distinguish cell-autonomous vs. non-autonomous effects due to InsR deficiency in neighboring hypothalamic cells, we reconstituted InsR specifically in POMC neurons (*L1-Pomc^+InsR^*). We further determined the developmental role of FoxO1 by POMC-specific ablation (*Pomc^–Foxo1^*). Our findings support a role of cell-autonomous InsR/FoxO1 signaling in determining the set point of ARC POMC cell number between puberty and young adulthood. Since each of these genetic models has attendant changes in systemic metabolism, it's possible that the hormonal and nutrient milieu contributes to alterations in POMC neuron development. However, we saw a decreased number of POMC cells in both *Pomc^–Foxo1^* (relative to wild type) and *L1-Pomc^+InsR^* (relative to L1), two models with opposite metabolic features: *Pomc^–Foxo1^* mice display hypophagia, hyperleptinemia, and normal insulin sensitivity [8], while *L1-Pomc^+InsR^* mice are hyperphagic, hypoleptinemic, and profoundly insulin resistant [7]. While these observations suggest that the role of POMC cell number is dependent on humoral and post-synaptic regulation of neuronal function, they also argue against a compensatory post-pubertal change in POMC cell number secondary to systemic cues.

The existing models cannot distinguish whether changes in POMC neuron number arise from changes in neuronal function subsequent to initial differentiation or from alterations in the rates of POMC cell birth (i.e. recruitment from the pool of NSCs) and death, as rates of cell death and *de novo* neurogenesis are generally low in the mediobasal hypothalamus [16], [25] and difficult to assess in a cross-sectional study and under non-stimulated conditions. However, FoxO1 has pro-apoptotic activities in different cell types and promotes cell death in postmitotic granule neurons [26], suggesting that the FoxO1-dependent increase in POMC neuron number in our study is independent of cell death. In addition, although insulin hasn't been previously linked to neurogenesis, studies have shown that insulin-like growth factor I (IGF-I)-dependent neurogenesis in adult rats did not affect any particular nucleus and occurred diffusely throughout the hypothalamus, with the exception of the periventricular zone of the so-called overlapping region (considered a neurogenic niche) [27]. Additional lineage tracing studies using reporter genes will be necessary to distinguish the potential effects of InsR/FoxO1 signaling on NSC-derived neurogenesis *vs.* functional plasticity.

Of note, POMC neuron number at Bregma −1.4 to −1.5 mm appears especially sensitive to alterations in InsR/FoxO1 signaling, suggesting that this region of the ARC harbors a functionally distinct sub-population of POMC neurons. A number of studies have reported that POMC neurons at different rostrocaudal or mediolateral coordinates can have different repertoires of transcription factors and hormone/neuropeptide responsiveness [28], [29], [30], [31]. A recent study showed that only a subset of POMC neurons express InsR, with those in mid-ARC having higher prevalence of InsR expression (36%) than those in rostral (26%) or caudal ARC (9%) [32], consistent with the localization of InsR/FoxO1-sensitive POMC neurons in the current study. Although we did not analyze InsR or FoxO1 expression in different ARC regions, it's reasonable to assume that the POMC neurons affected by the knockout and knock-in of InsR and FoxO1 are the most insulin-responsive.

We also uncovered a novel phenomenon of aging-associated decline in ARC POMC neurons between 15 and 30 weeks of age (data not shown). This process occurs independently of InsR/FoxO1 signaling in POMC neurons, as it's not affected in L1 or *Pomc^–Foxo1^* mice. Neurotrophic factors such as progranulin [33] and brain-derived neurotrophic factor (BDNF) [34] decrease in the hypothalamus with aging and have been suggested to contribute to the reduced plasticity of this region observed in older rodents. Notably, BDNF infusion into the brain suppressed hyperphagia in adult mice with deficient melanocortin-4 receptor signaling, although ARC POMC neurons were not directly examined [35]. In addition, ectopic expression of the neuron survival factor, glial cell line-derived neurotrophic factor, by way of adeno-associated virus, reduced food intake and decreased aging-related obesity in adult rats, associated with activation of corticotropin-releasing factor neurons in the paraventricular nucleus [36], consistent with increased melanocortin tone. Additional studies will be required to determine the contribution of POMC neurons to these effects and the functional significance of the decline in POMC cell number in aging-associated obesity.

## Materials and Methods

### Mice

C57BL/6 mice were obtained from Jackson Laboratories, ME, USA. *Insr^−/−^* [37], *Ttr-INSR* transgenic, *βac/INSR* knock-in [18], *Foxo1^flox/flox^* [38], and *Pomc-Cre* mice [39] have been previously described. *Insr^+/−^::Ttr-INSR::βac/INSR* mice were intercrossed with *Pomc-Cre* mice to generate *Insr^−/−^::Ttr-INSR::βac/INSR::Pomc-Cre* (*L1-Pomc^+InsR^*) mice [7]. WT littermates were used as controls. POMC-specific FoxO1 knockouts (*PomcCre.Foxo1^lox/lox^*: *Pomc^–Foxo1^*) and wild type control mice (*PomcCre.Foxo1^+/+^*, *Foxo1^lox/+^*, or *Foxo1^lox/lox^*) were generated by mating *Pomc*(Cre) with *Foxo1^lox/lox^* mice on a mixed 129sv×C57B/6 background [8]. Genotyping was performed as previously described [18], [39], [40], [41].

Due to stochastic embryonic expression of *Pomc*(Cre), ∼5% of the offspring mice showed widespread recombination and were excluded from analysis. To exclude possible effects related to sexual dimorphism or estrogenic effects on the melanocortin system [42], only male mice were included in the analyses.

Depending on the genotype, 9–23% of the offspring on the L1 background developed diabetes in early adulthood. All mice with a diabetic phenotype were excluded from the analyses. Non-diabetic mice of both genotypes similarly displayed modest glucose intolerance and unaltered insulin tolerance test results as compared to wild type controls [7].

Animal procedures were conducted in compliance with Columbia University Institutional Animal Care and Utilization Committee protocols in accordance with NIH guidelines; the study was approved by the Columbia University Institutional Animal Care and Utilization Committee (permit number AAAA9322). Mice were housed in groups of 3–5 at 22–24°C on a 12-hr light/dark cycle. Animals had *ad libitum* access to water and food (normal chow diet containing 62.1% calories from carbohydrates, 24.6% from protein, and 13.2% from fat; PicoLab rodent diet 20, #5053; Purina Mills, LLC, MO, USA).

### Fluorescent *in situ* hybridization (FISH) and neuron quantification

All animals were fed ad libitum on normal chow diet and sacrificed 4–6 hours after lights-on. Mice were anaesthetized and perfused transcardially with saline, followed by 4% paraformaldehyde in phosphate buffer (for FISH). Brains were dissected, post-fixed, equilibrated in 30% sucrose, and frozen in OCT (Sakura, CA, USA). Serial 10 µm-thick coronal sections were prepared from bregma −0.8 mm to −2.4 mm. *Pomc* and *Npy* riboprobes were synthesized using T7 RNA polymerase and DIG labeling mix (Roche, IN, USA). In situ hybridization using tyramide signal amplification reagents (PerkinElmer, MA, USA) and nuclear staining with Hoechst 33342 (Molecular Probes/Invitrogen) were performed on every tenth section throughout the ARC. Pictures were taken, and neurons with *Pomc* or *Npy* expression were counted and marked digitally using Adobe Photoshop to prevent multiple counts as described [43]. Results are expressed as counts of *Pomc*-positive, nucleus-containing cell bodies per hemisection (average of the left and the right) or the sum of these cells in all nine counted sections of the ARC (Bregma −1.1 mm to −1.9 mm).

### Statistical Methods

Data sets were analyzed for statistical significance with Student's t-test. Homogeneity of variances was tested by F-Test, and resulting values compared to the critical values of the F distribution table at the respective α levels. Accordingly, homoscedastic or heteroscedastic unpaired t-test was performed in the case of homogeneous or nonhomogeneous variances, respectively. The threshold for statistical significance was set at P<0.05.

## Supporting Information

Figure S1 **Unaltered** ***Pomc*** **neuron number in neonatal and pre-pubertal L1 mice.** Number of *Pomc*-expressing cells per ARC hemisection in L1 mice (grey bars) and WT controls (white bars) at postnatal day 7 (**A**) and at the age of 35 days (**B**). n = 4–8. All data are means ± S.E.M.
(TIF)

Figure S2 **Number of** ***Pomc*** **-positive ARC cells in adult L1 and** ***L1-Pomc^+InsR^*** **mice.** Total number of *Pomc*-positive cells counts per hemi-ARC in L1 mice (grey symbol, n = 5), *L1-Pomc^+InsR^* mice (yellow symbol, n = 6) and WT controls (white symbol, n = 5) at the age of 15 weeks. All data are means ± S.E.M.
(TIF)

Figure S3 **Unaltered** ***Pomc*** **neuron number in neonatal and pre-pubertal** ***Pomc^–Foxo1^*** **mice.** Number of *Pomc*-expressing cells per ARC hemisection in *Pomc^–Foxo1^* mice (black bars) and the respective WT controls (white bars) at postnatal day 7 (**A**) and at the age of 35 days (**B**). n = 4–5. All data are means ± S.E.M.
(TIF)

Figure S4 **Number of** ***Pomc*** **-positive ARC cells in adult** ***Pomc^–Foxo1^*** **mice.** Total number of *Pomc*-positive cell counts per hemi-ARC in *Pomc^–Foxo1^* mice (black bar, n = 9), and WT controls (white bar, n = 9) at the age of 15 weeks. All data are means ± S.E.M.
(TIF)
