# Supplementary figures and images for: InsR/FoxO1 Signaling Curtails Hypothalamic POMC Neuron Number
Source: PLoS One. 2012 Feb 2;7(2):e31487. doi: 10.1371/journal.pone.0031487 (PMC3271107; doi:10.1371/journal.pone.0031487)

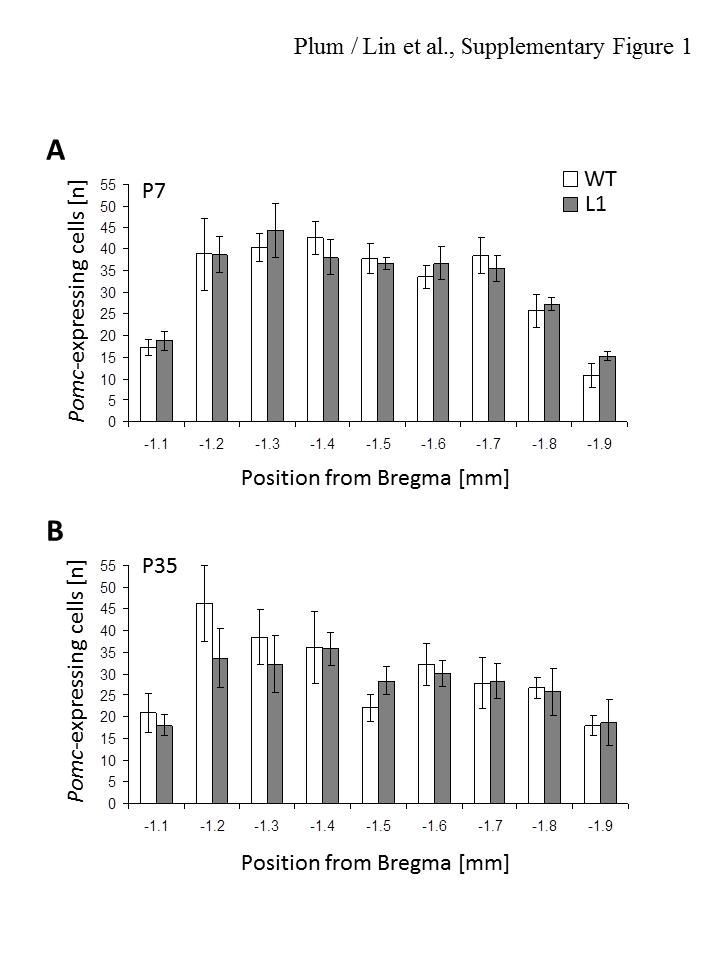

Supplement: Figure S1 — Unaltered Pomc neuron number in neonatal and pre-pubertal L1 mice. Number of Pomc-expressing cells per ARC hemisection in L1 mice (grey bars) and WT controls (white bars) at postnatal day 7 (A) and at the age of 35 days (B). n = 4–8. All data are means ± S.E.M. (TIF) [file pone.0031487.s001.tif]

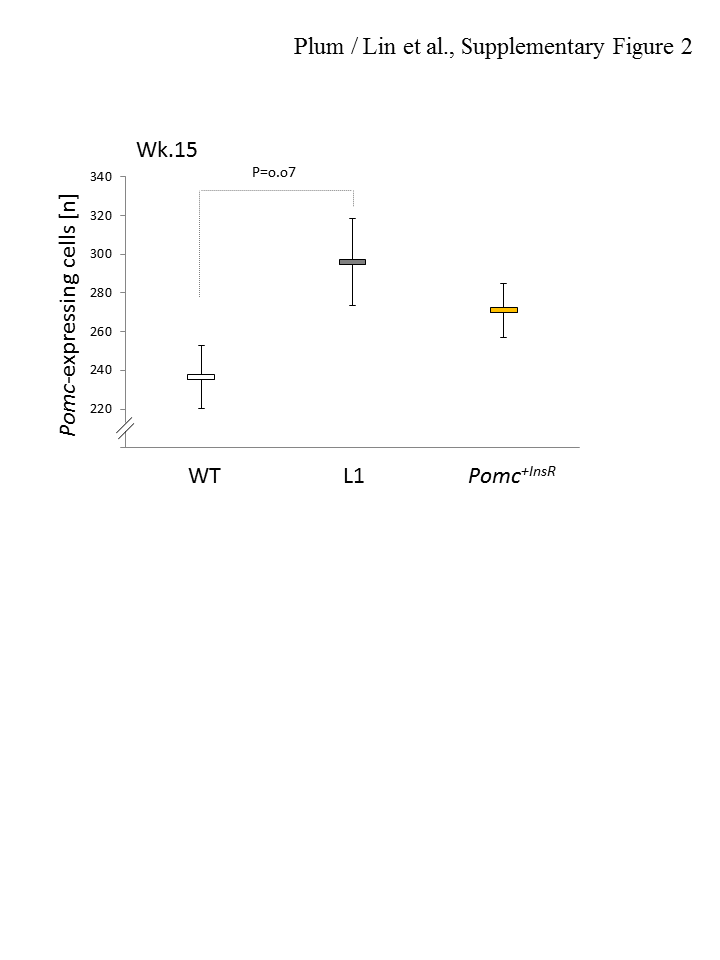

Supplement: Figure S2 — Number of Pomc -positive ARC cells in adult L1 and L1-Pomc+InsR mice. Total number of Pomc-positive cells counts per hemi-ARC in L1 mice (grey symbol, n = 5), L1-Pomc+InsR mice (yellow symbol, n = 6) and WT controls (white symbol, n = 5) at the age of 15 weeks. All data are means ± S.E.M. (TIF) [file pone.0031487.s002.tif]

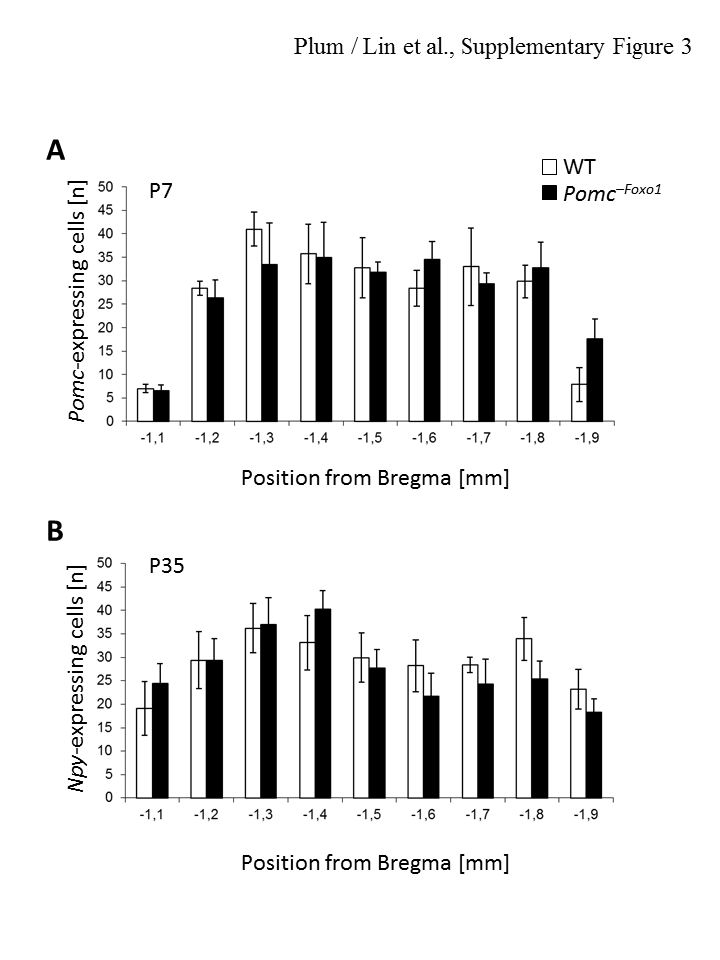

Supplement: Figure S3 — Unaltered Pomc neuron number in neonatal and pre-pubertal Pomc–Foxo1 mice. Number of Pomc-expressing cells per ARC hemisection in Pomc–Foxo1 mice (black bars) and the respective WT controls (white bars) at postnatal day 7 (A) and at the age of 35 days (B). n = 4–5. All data are means ± S.E.M. (TIF) [file pone.0031487.s003.tif]

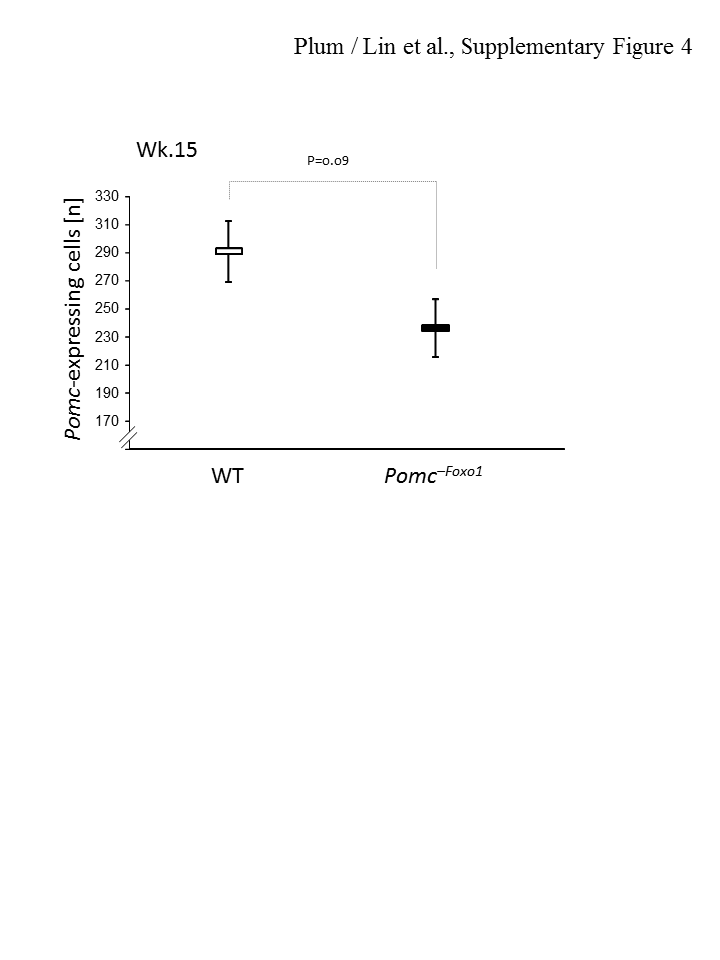

Supplement: Figure S4 — Number of Pomc -positive ARC cells in adult Pomc–Foxo1 mice. Total number of Pomc-positive cell counts per hemi-ARC in Pomc–Foxo1 mice (black bar, n = 9), and WT controls (white bar, n = 9) at the age of 15 weeks. All data are means ± S.E.M. (TIF) [file pone.0031487.s004.tif]
